# Supplementary material for: Atomic Magnetometer Achieves Visual Salience Analysis in Drosophila
Source: Sensors (Basel). 2023 Jan 17;23(3):1092. doi: 10.3390/s23031092 (PMC9921713; doi:10.3390/s23031092)
Supplement: Supplementary file 1 [file sensors-23-01092-s001.zip › sensors-2151062-supplementary.pdf]

Atomic magnetometer achieves visual salience analysis in Drosophila

Authors:

Fan Liu †, Dongmei Li †, Yixiao Li, Zhao Xiang, Yuhai Chen, Zhenyuan Xu, Qiang Lin, Yi Ruan *

Zhejiang Provincial Key Laboratory and Collaborative Innovation Center for Quantum Precision Measurement, College of Science, Zhejiang University of Technology, Hangzhou 310023, China

* Correspondence: yiruan@zjut.edu.cn

† These authors contributed equally to this work.

This PDF file includes:

Supplementary information Text

Figs. S1 to S4

Spin exchange relaxation free atomic magnetometer (SERF AM):

A single-light spin exchange relaxation free atomic magnetometer(SERF AM) achieved $20\,fT/\sqrt{Hz}$ before measuring, and the noise power spectral density described the sensitivity as shown in FIG.S1.

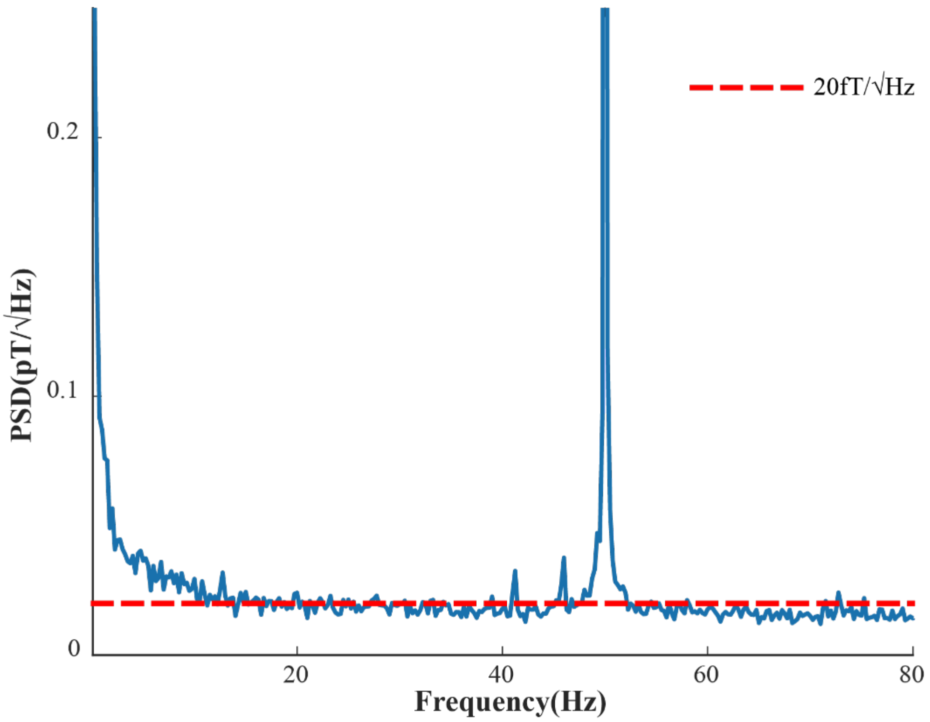

Figure S1: Sensitivity of SERF atomic magnetometer

Figure S2 shows that the PSD in the frequency range between 1 and 30 Hz was significantly increased under dead Drosophila condition compared with unloading in our measurements. It should be noted that such a raise of PSD under dead drosophila condition may be caused by the unconscious muscle movement of the body after the brain of the fruit fly dies.

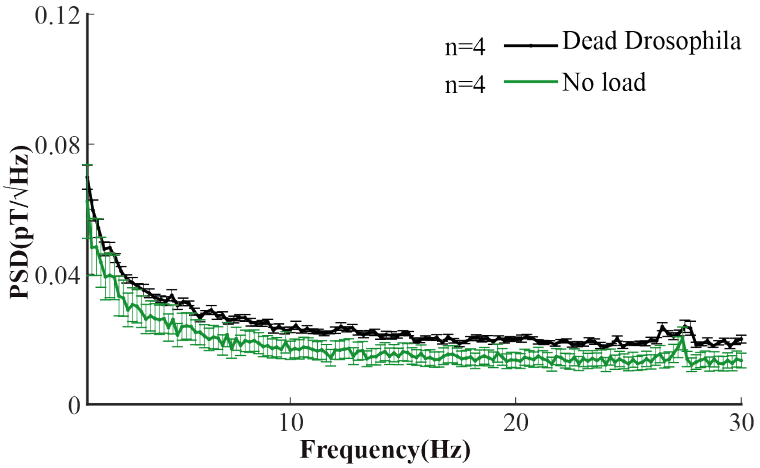

Figure S2: Power Spectral Density (PSD) between 1 - 30 Hz, dead flies (black line) and unloading (green line).

As indicated in Fig S3 and Fig S4, that 1 - 20 Hz and 30 - 80 response in the presence of stimulation have almost no change compared with that without stimulation $p > 0.05$, $n = 11$ flies.

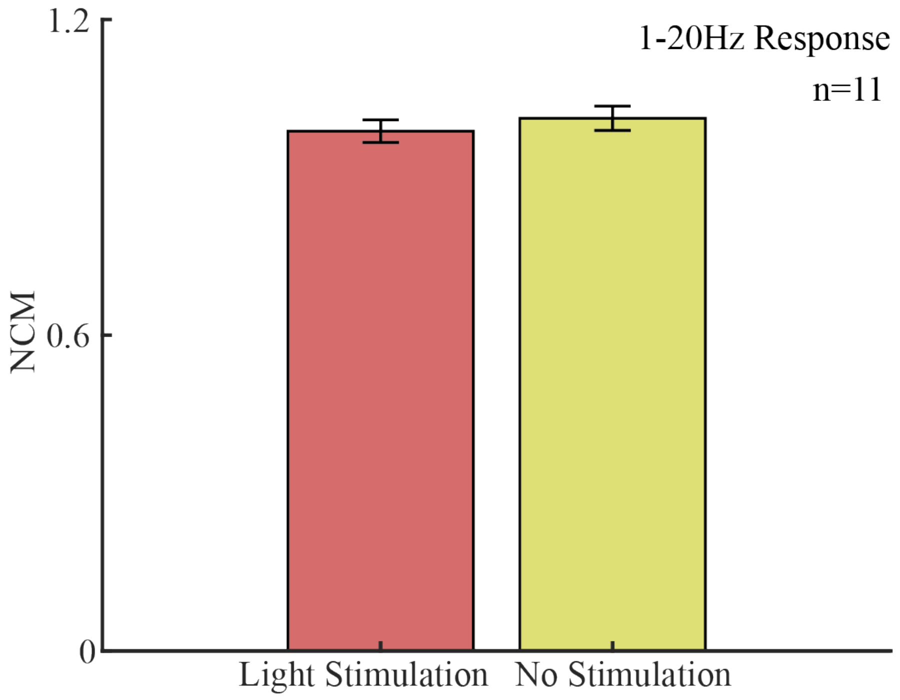

Figure S3: Average 1-20 Hz NCM (±SEM) : No Stimulation (yellow) , Light Stimulation (red).

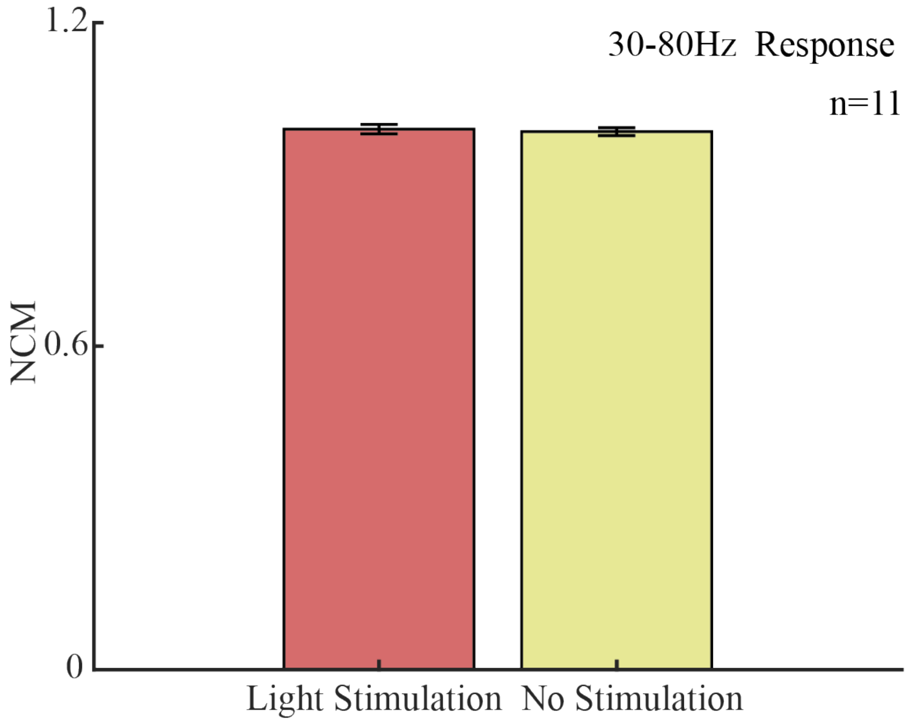

Figure S4: Average 30-80 Hz NCM (±SEM): No Stimulation (yellow), Light Stimulation (red).
